# Giant photovoltaic effect of ferroelectric domain walls in perovskite single crystals

**DOI:** 10.1038/srep14741

**Published:** 2015-10-07

**Authors:** Ryotaro Inoue, Shotaro Ishikawa, Ryota Imura, Yuuki Kitanaka, Takeshi Oguchi, Yuji Noguchi, Masaru Miyayama

**Affiliations:** 1 Dept. of Applied Chemistry, School of Engineering, The University of Tokyo, Bunkyo-ku, Tokyo 113-8654, Japan

## Abstract

The photovoltaic (PV) effect in polar materials offers great potential for light-energy conversion that generates a voltage beyond the bandgap limit of present semiconductor-based solar cells. Ferroelectrics have received renewed attention because of the ability to deliver a high voltage in the presence of ferroelastic domain walls (DWs). In recent years, there has been considerable debate over the impact of the DWs on the PV effects, owing to lack of information on the bulk PV tensor of host ferroelectrics. In this article, we provide the first direct evidence of an unusually large PV response induced by ferroelastic DWs—termed ‘DW’-PV effect. The precise estimation of the bulk PV tensor in single crystals of barium titanate enables us to quantify the giant PV effect driven by 90° DWs. We show that the DW-PV effect arises from an effective electric field consisting of a potential step and a local PV component in the 90° DW region. This work offers a starting point for further investigation into the DW-PV effect of alternative systems and opens a reliable route for enhancing the PV properties in ferroelectrics based on the engineering of domain structures in either bulk or thin-film form.

The photovoltaic (PV) effect in polar materials has attracted substantial interest, because the photoconversion mechanism can be exploited for the development of advanced solar cells that generate a high voltage. The bulk PV effect has been extensively studied in ferroelectric oxides^1^,^2^,^3^,^4^,^5^,^6^,^7^,^8^, compound semiconductors^9^,^10^ and fluoride polymers^11^. The introduction of transition-metal atoms into the host lattices has been shown to be effective in enhancing the bulk PV effect under visible-light irradiation, because defect states in the bandgap result in light absorption and the subsequent charge separation^5^,^12^.

Recent studies on ferroelectric thin films have demonstrated that bismuth ferrite (BiFeO_3_: BFO) with ferroelastic domain walls (DWs) delivers above-bandgap voltages that can be tuned by the number of the DWs^13^. An internal quantum efficiency in the DWs has been reported as high as 10%^14^. The microscopic origin of the high photovoltage is shown to originate from an electrostatic potential step at the DWs. Meanwhile, the temperature-dependent PV studies have revealed that BFO films generate a high photovoltage by controlling the conductivity of the DWs^15^. This anomalous PV effect is thought to be due to the bulk PV effect, not to the electrostatic potential step at the DWs. Essentially, the bulk PV effect arises from spatial symmetry breaking in polar materials^16^,^17^ and can be described in terms of the bulk PV tensor^18^. Recent theoretical calculations^19^,^20^,^21^,^22^ and atomic-scale microscopy^23^,^24^ have shown that spatial symmetry breaking is preserved in the local region of the ferroelastic DWs. These studies suggest that the DW region inherently has a local PV component similar to the bulk PV effect in addition to the electrostatic potential step.

Until now, there has been considerable debate over the mechanism of the PV effects in ferroelectrics in the presence of ferroelastic DWs, owing to lack of information on the bulk PV tensor of the host crystals. In this article, we present the first direct evidence that ferroelastic DWs deliver an anomalously large PV response in a perovskite ferroelectric crystal. We term it the ‘DW’-PV effect. We select barium titanate (BaTiO_3_: BT) as a model system to investigate these effects. The precise estimation of the bulk PV tensor allows us to quantify the contribution of 90° DWs in BT single crystals, revealing that the field strength due to the DW-PV effect is far beyond the bulk PV effect. We show that this extremely large field stems from an effective electric field consisting of a potential step and a local PV component in the 90° DW region.

## Results

**Figure 1:**
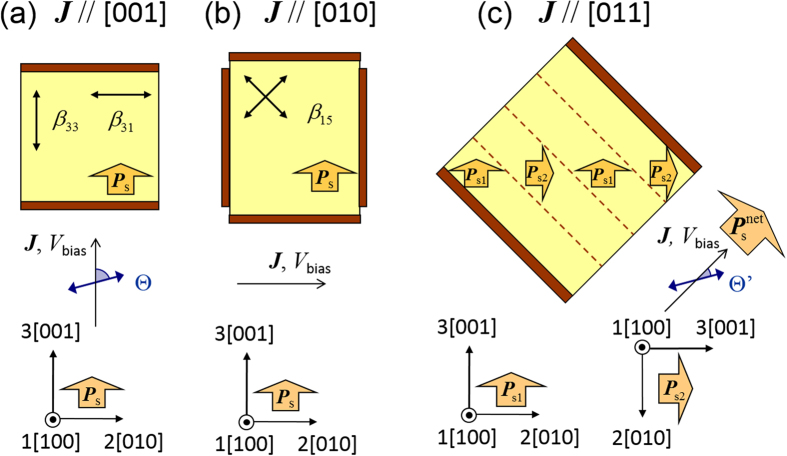
Electrode configurations with respect to the crystal axes: (**a**) *J*//[001], (**b**) *J*//[010] and (**c**) *J*//[011]. The light-polarizations (Θ and 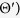 are defined as the angles between the polarization plane of light and the measured direction of ***J***. The two-headed black arrows written with the components of bulk PV tensor (*β*_33_, *β*_31_ and *β*_15_) represent the light-polarization of the corresponding components.

We evaluated the PV properties of the single crystals of Mn-doped BT (Mn-BT) in three different configurations shown in Fig. 1. The electronic mechanism of the photocurrent properties under visible-light irradiation in Mn-BT has been reported in ref.^12^. Here, we focus on the impact of 90° DWs on the PV properties. Throughout this paper, we denote photocurrent density vector by ***J***, bias voltage by *V*_bias_. We define short-circuit current density (*J*_SC_) as the *J* value at *V*_bias_ = 0 and open-circuit voltage (*V*_OC_) as the *V*_bias_ value at *J* = 0.

### *J - V*_bias_ characteristics

**Figure 2:**
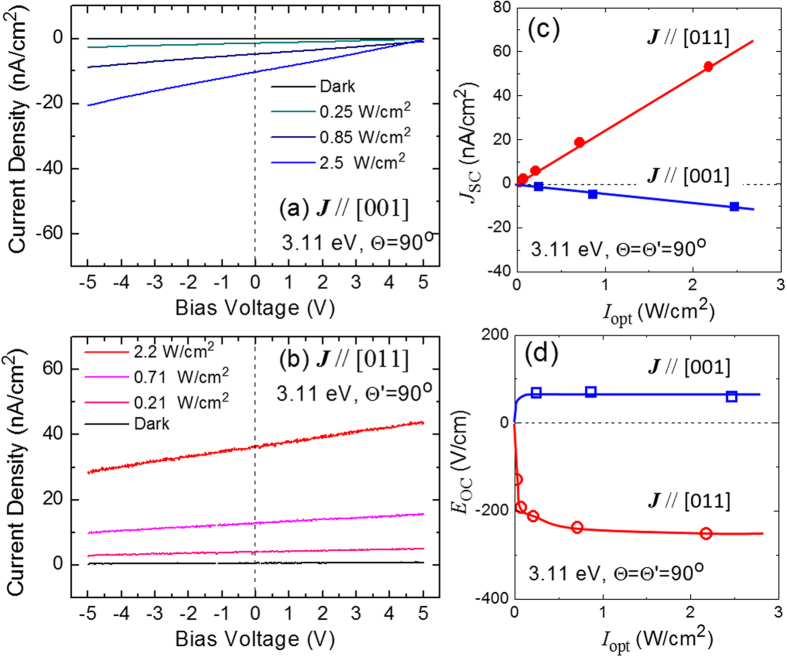
Current density - voltage characteristics of the Mn-doped BT samples in (**a**) the *J*//[001] and (**b**) the *J*//[011] configurations. The light-polarization is fixed perpendicular to the measurement directions of photocurrent (Θ = 90°, 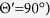. (**c**) Short-circuit current density (*J*_SC_) as a function of light intensity 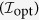. The solid lines are the linear fitting results. (**d**) Open-circuit electric field (*E*_OC_) as a function of 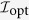. The solid curves are guides to the eye. Note that the signs of *J*_SC_ are opposite in the ***J***//[001] and the ***J***//[011] configurations.

Figure 2a-2b represent the *J* - *V*_bias_ characteristics of the Mn-BT samples in the ***J***//[001] and the ***J***//[011] configurations, respectively, under light irradiation (3.11 eV, Θ = 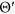 = 90°). In the both configurations, we confirmed a linear relation between *J* and *V*_bias_. It is worth noting that the signs of *J*_SC_ and of *V*_OC_ are different between the ***J***//[001] and the ***J***//[011] configurations. That is, the photocurrent flows in the direction *opposite* to the spontaneous polarization (***P***_s_) in the single-domain state (Fig. 2a) whereas the photocurrent is generated in the *same* direction as the *net* spontaneous polarization 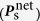 in the 90° domain structure (Fig. 2b).

In Fig. 2c-2d we plot the light intensity 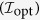 dependences of *J*_SC_ and open-circuit electric field (*E*_OC_). While *J*_SC_ is proportional to 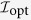, *E*_OC_ saturates in the high-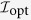 region above ~1 W/cm^2^. Hereafter we discuss this high-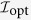 region, where the dark conductivity is negligible.

Values of the photoconductivity, 
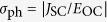
, estimated from the slope of the *J* - *V*_bias_ data (Fig. 2a,b) are proportional to 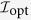 and the proportional constants 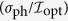 in both configurations are tabulated in Table 1. We note that 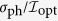 is almost the same in both the configurations. The 90° DWs that are present in the ***J***//[011] samples do not affect the overall behaviour of *σ*_ph_. This experimental result provides the fundamental basis for identifying the DW-PV effect, as described below.

### Light-polarization dependence of *J*_SC_

**Figure 3:**
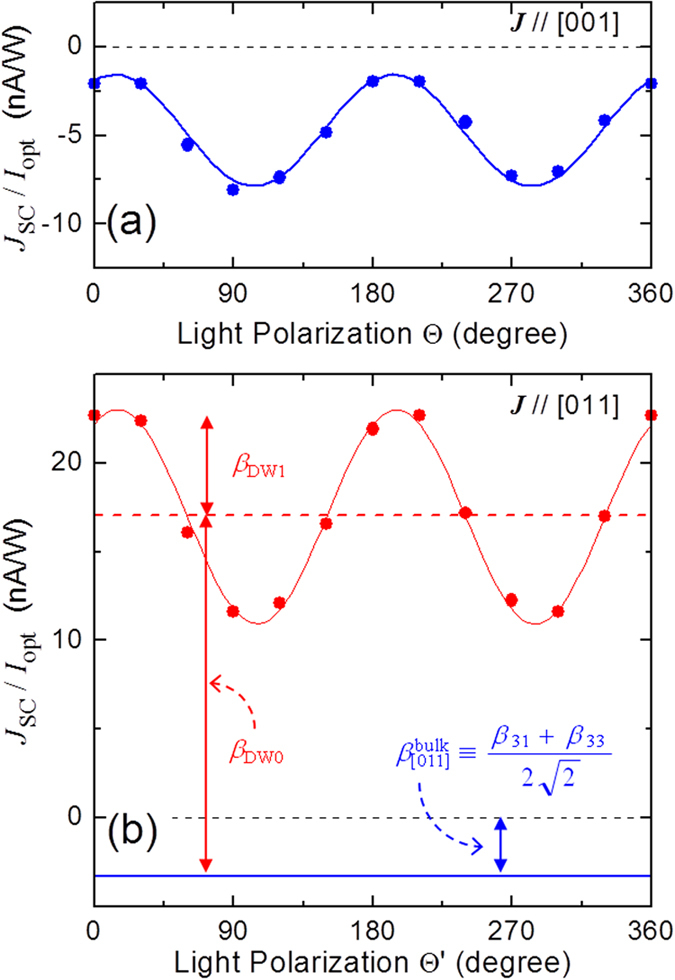
Photocurrent densities normalized by light intensity 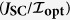 as a function of the light-polarization (Θ or 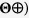 in (**a**) the *J*//[001] and (b) the *J*//[011] configurations. The photon energy (*hv*) is 3.11 eV. Solid lines denote the fitting results.

In Fig. 3 we plot the short-circuit photocurrent density normalized by the light intensity 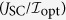 observed for the Mn-BT samples in the ***J***//[001] and the ***J***//[011] configurations. In both of the configurations we could confirm a strong dependence of *J*_SC_ on the light-polarization.

According to the bulk PV tensor in the tetragonal BT system [see Eq. (5) in Method], the photocurrent density in the ***J***//[001] configuration 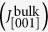 can be written by


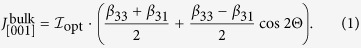


The fitting of the data shown in Fig. 3a leads to 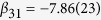 nA/W and 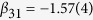 nA/W at 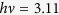 eV. The standard deviations of these parameters are shown in parenthesis and estimated to be ~5% at most.

From the results measured in the ***J***//[010] configuration (Fig. 1b) we conclude that 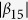 is smaller than the detection limit of our measurement system, i.e., ~3 pA/W. Since the 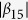 value of ~3 pA/W is two orders of magnitude smaller than those of other components 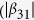 and 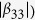, we neglect *β*_15_ throughout this paper.

In the ***J***//[011] configuration (Fig. 3b) we found that the photocurrent flows in the same direction as 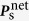, which cannot be explained by the bulk PV effect as described below. The poling in the ***J***//[011] configuration leads to a domain structure in which two kinds of spontaneous polarizations (***P***_s1_ and ***P***_s2_) with different orientations are present with the 90° DWs. The photocurrent density in the ***J***//[011] configuration arising from the bulk PV effect 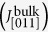 can be expressed by


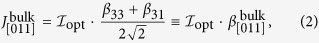


which is independent of the light-polarization 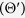. The derivation of Eq. (2) based on effective electric fields is given in Supplementary Information.

In Fig. 3b we also put the contribution of 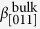 expected from Eq. (2). We note a considerable component of positive *J*_SC_ with a strong dependence on 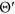, which goes beyond the bulk PV effect with a negative constant 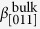. The experimental fact that 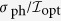 takes almost the same value regardless of the presence or absence of the DWs leads us to consider that the 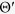-dependent, positive 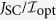 is not relevant to *σ*_ph_. These results strongly support the conclusion that the behaviour of *J*_SC_//[011] does originate from the 90° DWs. The thickness (*w*_DW_) of the 90° DW region is reported to be 2–100 nm^25^,^26^,^27^,^28^,^29^,^30^. Since *w*_DW_ is two to three orders of magnitude smaller than the DW spacing (*W* ~ 15 *μ*m), the large value of *J*_SC_//[011] appears to arise from a giant PV effect in the local region of the 90° DWs.

We define 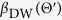 as the difference between the measured 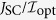 and the bulk PV effect 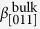. Using the following functional form: 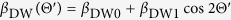, where *β*_DW0_ and *β*_DW1_ correspond to the positive offset and the amplitude, respectively, the fitting yields 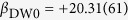 nA/W and 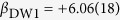 nA/W. We emphasize that 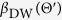 is not calculated locally in the DW region but is averaged over the entire samples.

### The bulk PV effect vs. the DW-PV effect

**Figure 4:**
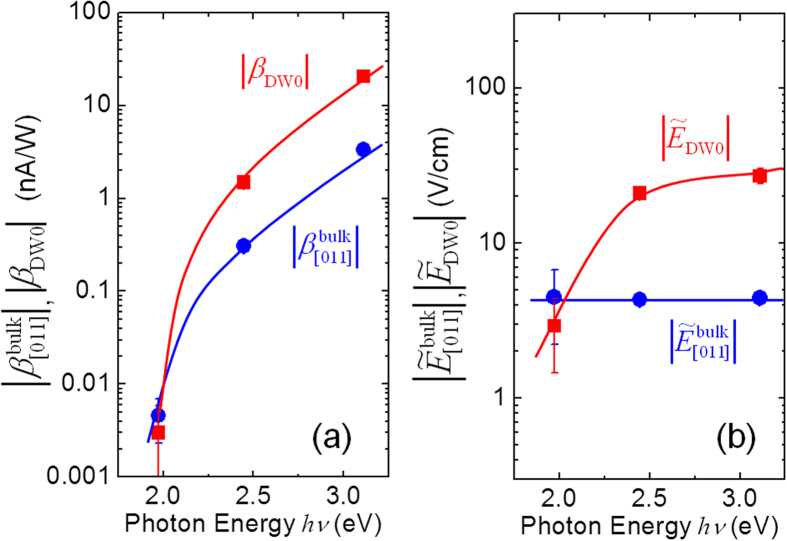
Comparison of bulk PV effect with DW-PV effect of Mn-BT: (**a**) 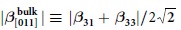 and 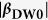, (**b**) corresponding effective electric fields 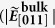 and 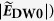. The solid curves are guides to the eye. The standard deviation of each data point is shown as an error bar.

The results measured at three wavelengths (λ = 405, 515 and 639 nm) are summarized in Table 2. Here we focus on the positive offset 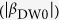 and the bulk PV effect 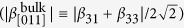 of the Mn-BT samples, which are plotted as a function of *hv* in Fig. 4a. Except for the data at *hv* = 1.97 eV, which are comparable to the detection limit of ~3 pA/W, we note that 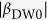 is 5–10 times as large as 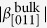.

As described above, we found that *σ*_ph_ does depend neither on the light-polarization nor on the crystal orientation nor on the presence/absence of the DWs. We can thus express *J*_SC_ using effective electric field 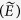 as 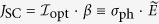 and define the effective electric field 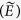 for representing the PV effect by


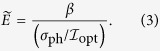


Here 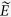 is equivalent to 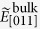 for the bulk PV effect and to 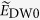 for the DW-PV effect, where *β* corresponds to its respective 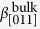 and *β*_DW0_. Considering the linear current - voltage characteristics and the independence of 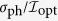 on 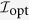, 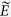 is identical to the open-circuit electric field (*E*_OC_).

In Fig. 4b we plot the 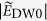 and 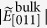 values as a function of *hv*. We found that 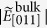 does not depend on *hv*, which seems to be a specific feature of the bulk PV effect. Even though the DW-PV effect occurs in an extremely small volume only in the 90° DW region, the resultant effective field averaged over the entire samples 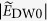 is large compared with 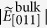. These experimental results provide direct evidence that the 90° DWs deliver a giant PV effect. Taking into account that 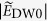 is significantly small at 1.97 eV and that 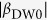 indicates a sharp decrease in the *hv* range of 2.0–2.5 eV, we speculate that the DW-PV effect due to *β*_DW0_ is activated at above a threshold of *hv*, the reason of which is still under investigation.

## Discussion

The bulk PV effect stems from an asymmetry in the photogenerated carrier dynamics in polar materials and can be interpreted in terms of effective electric fields. We introduce a single parameter (*γ*) representing the asymmetry in the photogenerated carrier density and relate the effective electric field 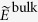 ( = *J*_SC_/*σ*_ph_) with *γ* as


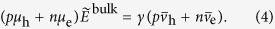


Here *n* and *p* denote electron density and hole density, *μ*_e_ and *μ*_h_ their mobilities. We also define 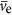 and 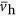 as the averaged drift velocities projected onto the ***P***_s_ direction where the average is taken over a solid angle of 2*π*. The derivation of Eq. (4) is given in Supplementary Information. As shown in Fig. 4b 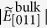 is almost independent of *hv*. Based on this result it is reasonable to assume that in the [011] configuration the *γ* value does not depend on *hv* even though 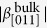 and *σ*_ph_ are strongly dependent on *hv*.

The carrier dynamics under steady-state conditions can be interpreted in terms of electrochemical potential^31^. We first discuss the effective electric field 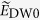 arising from the DW-PV effect using the electrochemical potential gradient. Noting that 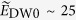 V/cm is averaged over the entire samples with the 90° domain structure (*W*~ 15 *μ*m), we can regard the net photovoltage per DW 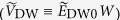 as ~37.5 mV. The 90° DW region indeed has a significant volume with a *w*_DW_ of 2–100 nm^25^,^26^,^27^,^28^,^29^,^30^. Adopting *w*_DW_ ~ 10 nm as a representative value, we estimate the corresponding effective electric field in the DW region, 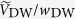, to be ~37.5 kV/cm. This field strength is quite large, i.e., 8000–8500 times as large as that inside the domains 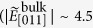 V/cm).

One of the key factors affecting 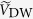 in the 90° DW region is electrostatic potential step 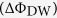
20. A rotation of the ***P***_s_ vector in the 90° DW region is accompanied by 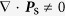
22. The variation in ***P***_s_ normal to the DW results in an electric double layer, yielding 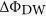 at each of the DW. Another factor affecting 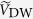 is a local PV component peculiar to the 90° DW region. The DW region has the non-centrosymmetric nature, i.e., a ferroelectric polarization to a considerable degree. The substantial strain in the 90° DW region with 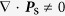 forces us to consider a local PV component, which appears to be greatly different from those inside the domain, i.e., from the bulk PV tensor. In fact, the large dependence of 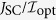 on the light-polarization shown in Fig. 3b (corresponding to *β*_DW1_) is not predicted by 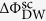. Furthermore, the experimental fact of the large *β*_DW0_ value with an oscillation due to *β*_DW1_ validates the local PV component of the 90° DW region, which is clearly distinct from the bulk PV effect. Therefore, we take into account the following two factors affecting 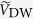 in the 90° DW region: 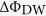 and the local PV component.

First we assessed the effect of 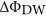 on 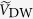. According to the first-principles calculations^20^, 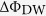 is estimated to be ~230 mV in the 90° DW in the tetragonal BT system. Under light irradiation, 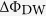 is partially screened by the photogenerated carriers. A detailed study including the screening of 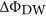 has been performed for BFO films based on a drift diffusion analysis^14^. We estimate the electrostatic potential step involving the screening effect, i.e., the screened electrostatic potential step 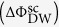 to be ~50 mV at least, which is still larger than the experimental value of 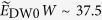 mV. Our estimation focusing on the carrier-density dependence of chemical potential is given in Supplementary Information.

Next we investigated the effect of the local PV component on 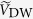. As described above, the non-centrosymmetric structure in the 90° DW region does produce the local PV component, which is superimposed on 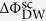. Assuming *w*_DW_ ~ 10 nm, we estimate the effective electric field originating from the local PV component in the 90° DW region to be 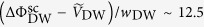 kV/cm.

We point out that the local PV component in the DW region can also explain the anomalous PV properties reported for BFO films. In the original report, Yang *et al.* have observed that *V*_OC_ increases in proportion to the number of the 71° DWs between electrodes^13^. They have proposed a model in which 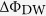 is the origin of the PV properties, together with the fact that a *V*_OC_ evaluated for each DW of ~10 mV is quite close to a potential step 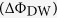 across the 71° DW of 20 mV^14^,^21^. In contrast, Bhatnagar *et al.* have observed that *V*_OC_ markedly increases at low temperatures and that *J*_SC_ depends on the light-polarization, both of which cannot be explained only by 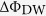. The behaviour of *V*_OC_ and *J*_SC_ is due not to 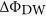 but to the bulk PV effect, and the bulk PV tensor was evaluated from the sinusoidal components in 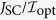
15. The data observed in 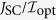 contain not only the sinusoidal component but also an apparently significant constant term. In their analysis, this constant term is thought to be caused by the combined effect of the experimental misalignments and is not taken into consideration. We infer that the DW-PV effect also contributes to the observed 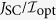 behaviour. The DW-PV effect involving 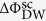 and the local PV component provides a reasonable explanation for the PV data reported for the BFO films, which are associated with both the number of DWs on the one hand^13^ and the bulk PV nature (the strong light-polarization dependence) on the other^15^.

**Figure 5:**
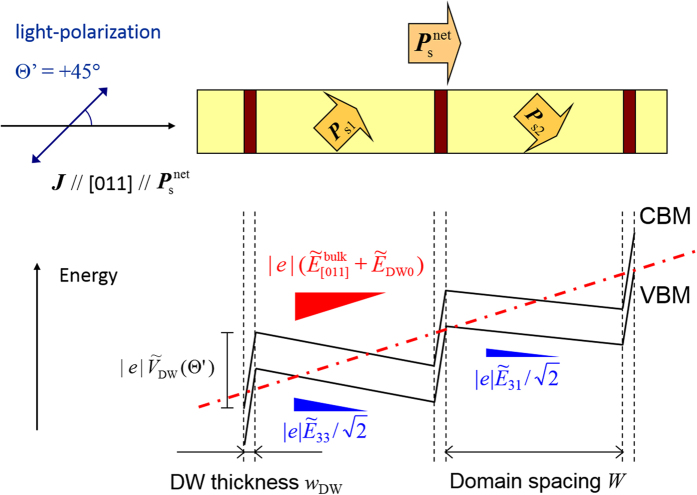
Schematic diagram of the valence band maximum (VBM) and conduction band minimum (CBM) taking the bulk PV and DW-PV effects into account under the open-circuit condition. The light-polarization angle 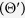 is fixed to +45°. The slopes inside the two types of alternate-stacking domains correspond to the effective electric fields, 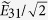 and 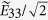. The effective electric field of the DW-PV effect 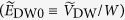 has a larger magnitude with the opposite sign than that of (averaged) bulk PV effect 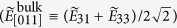. The direction of the short-circuit photocurrent is reversed by introducing the 90° domain structure.

Finally, we discuss why the DW-PV effect delivers a large positive photocurrent going beyond the negative bulk PV effect, i.e., 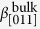. Figure 5 depicts the schematic diagram of valence band maximum (VBM) and conduction band minimum (CBM) under the open-circuit condition. The bulk PV and the DW-PV effects are incorporated as the effective electric fields. Although electronic band structures are modulated by the strain in the 90° DW regions, the modulation is assumed to be ~20% at most, as has been reported for 71° DW in BFO. Since the band modulation caused by the strain is much smaller than the influences of 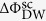 and the local PV component, we represent the CBM and the VBM as the two parallel segments. Given a light-polarization angle 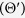 of +45°, the effective electric fields inside the domains are described as 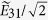 and 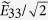, where 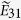 and 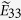 denotes those ascribed to *β*_31_ and *β*_33_, respectively. The electric field in each domain varies with the light-polarization angle 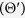 while the averaged field 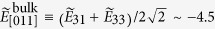 V/cm is independent of 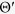. As described above, the effective electric field in the 90° DWs region 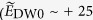 V/cm) and the DW spacing (*W* ~ 15 *μ*m) lead to the net photovoltage per DW 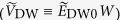 of ~37.5 mV, which is the sum of the 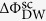 value and the local PV component. Noting that the magnitude of 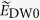
(positive) is larger than that of 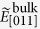
(negative), we estimate the net PV field in the [011] direction, 
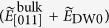
, to be ~+ 20.5 V/cm (the red dashed-dotted line in Fig. 5). Since the positive field arising from the DW-PV effect overcomes the negative field due to the bulk PV effect, the short-circuit current is reversed by the introduction of the 90° DWs.

We emphasize that the DW-PV effect can be assessed by examining the PV properties based on the precise estimation of the bulk PV tensor. Our report on the giant DW-PV effect opens a reliable route for enhancing the PV properties in ferroelectrics based on the engineering of domain structure in either bulk or thin-film form.

## Method

### Sample preparation

The samples were prepared from commercial BT single crystals (Neotron) and a Mn(0.25%)-doped BT bulk single crystal grown by a top-seeded solution growth method in our group. The image of the Mn-BT crystal is given in Supplementary Information. After cutting the crystals, we polished the top and bottom sides of the samples [the (100) and 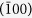 surfaces] and annealed them in air at 1250 °C for 12 h for recovery from mechanical damage incurred during the sample preparation. Electrodes were fabricated on the lateral sides by platinum sputtering. The poling was performed at an applied electric field of 2 kV/cm during a slow cooling from 150 °C down to room temperature through the Curie temperature (*T*_*C*_ ~ 130 °C).

### Electrode configurations

Figure 1 depicts the electrode configurations that we conducted the PV measurements. In all configurations, visible light was irradiated along the 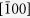 direction, which is perpendicular to the top surface. In the ***J***//[001] configuration (Fig. 1a) the electrodes on the (001) and 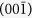 surfaces were used for the poling and the PV measurements. The photocurrent was measured along the [001] direction in the single-domain samples. In the ***J***//[010] configuration (Fig. 1b) the poling was performed along the [001] direction while the photocurrent was measured along the [010] direction. In the ***J***//[011] configuration (Fig. 1c) the electrodes on the (011) and 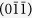 surfaces were used for the poling and the PV measurements. The poling yielded a 90° domain structure where two kinds of spontaneous polarizations (***P***_s1_ and ***P***_s2_) with different orientations are present. Using optical microscopy and piezoelectric force microscopy (PFM), we confirmed that the spacing between the 90° DWs was ~15 *μ*m for both the Mn-BT and BT samples. The typical PFM image of the domain structure is given in Supplementary Information. The photocurrent was measured along the direction of the net spontaneous polarization 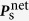, i.e., the [011] direction.

### PV measurements

The PV properties were measured at 25 °C. We denote photocurrent density vector by ***J*** and bias voltage by *V*_bias_. We measured the current - voltage characteristics under visible light irradiation with an intensity of 0.03–3 W/cm^2^ using three monochromatic laser modules [wavelength (photon energy *hv*); 405 nm (3.11 eV), 515 nm (2.45 eV) and 639 nm (1.97 eV)]. In the measurement of the PV properties, we excluded a transient current due to the capacitance and resistance of the samples. We also confirmed that currents arising from both the pyroelectric and piezoelectric effect are eliminated thoroughly under the measurement conditions in the steady state.

As indicated by the arrows in Fig. 1, the positive direction of ***J*** and *V*_bias_ is defined as that of the *net* spontaneous polarization, ***P***_s_ (Fig. 1a,b) or 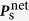
(Fig. 1c). In Fig. 2a,b the measured current density (*J*) is plotted as a function of *V*_bias_. We define short-circuit current density (*J*_SC_) as the *J* value at *V*_bias_ = 0 and open-circuit voltage (*V*_OC_) as the *V*_bias_ value at *J* = 0. In determining *V*_OC_ we extrapolated the linear *J* - *V*_bias_ characteristics in a limited range owing to the current amplifier used in our study. It is noteworthy that the positive/negative of *V*_OC_ means the opposite/same direction of the photovoltage generated inside the samples, because the open-circuit condition is achieved when *V*_bias_ cancels *V*_OC_ under light irradiation.

We define light intensity 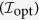 as the light power per unit area and denote open-circuit electric field (*E*_OC_) by the open-circuit voltage (*V*_OC_) divided by the electrode spacing. The light polarization was controlled by a half-wavelength plate and a polarizer. We represent the light-polarization (Θ or 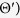 as the angle between the polarization plane of light and the measured direction of ***J***, as illustrated in Fig. 1.

### Tensor representation of the bulk PV effect

We describe the tensor representation of ***J*** arising from the bulk PV effect in the BT system. The tetragonal phase of BT with 4 mm point group symmetry has the bulk PV tensor with three independent non-zero components under the irradiation of linearly polarized light. We define the incident direction of light (// 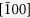 ) as *i* = 1 and the polar axis (//[001]) as *i* = 3. Using the polarization unit vector [***e*** = (*e*_1_, *e*_2_, *e*_3_)], the three non-zero components of the bulk PV tensor (*β*_31_, *β*_33_ and *β*_15_) and 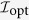, the photocurrent density vector [***J*** = (*J*_1_, *J*_2_, *J*_3_)] can be written as follows:


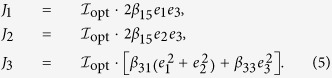


Taking into account that the bulk PV tensor (*β*_*ijk*_) is a third-rank tensor and is symmetric for the latter two indices (*j* and *k*), we adopt here the standard 3 × 6 matrix notation: 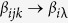 with 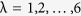, as used in the representation of the piezoelectric tensor.

## Additional Information

**How to cite this article**: Inoue, R. *et al.* Giant photovoltaic effect of ferroelectric domain walls in perovskite single crystals. *Sci. Rep.* **5**, 14741; doi: 10.1038/srep14741 (2015).

## Supplementary Material

Supplementary Information

## Figures and Tables

**Table 1:** Current density - voltage characteristics of the Mn-doped BT samples in the *J*//[001] and the *J*//[011] configurations under light irradiation (*hv* = 3.11 eV, Θ = Θ′ = 90°).

	*J*//[001]	*J*//[011]
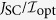 (nA/W)	−4.2	+15.8
*E*_OC_ (V/cm)	+59.1	−254
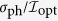 (pS cm/W)	76	68

The photoconductivity (*σ*_ph_) is defined as 
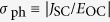
.

**Table 2:** The non-zero components of the bulk PV tensor (*β*_31_ and *β*_33_) and the coefficients of the DW-PV effect (*β*_DW0_ and *β*_DW1_).

	Mn-BT			BT	
	405 nm(3.11 eV)	515 nm(2.45 eV)	639 nm(1.97 eV)	405 nm(3.11 eV)	515 nm(2.45 eV)
*β*_31_ (nA/W)	−7.86(23)	−0.70(3)	−0.003(3)	−0.116(5)	−0.013(3)
*β*_33_ (nA/W)	−1.57(4)	−0.17(1)	−0.010(3)	−0.004(3)	−0.003(3)
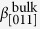 (nA/W)	−3.33(28)	−0.31(4)	−0.005(6)	−0.043(8)	−0.006(5)
*β*_DW0_ (nA/W)	+20.34(61)	+1.47(4)	+0.003(3)	+0.48(2)	+0.013(3)
*β*_DW1_ (nA/W)	+6.06(18)	+0.74(3)	+0.003(3)	+0.27(1)	+0.003(3)

The calculated values of 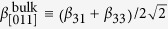 are also shown. For the DW-PV effect, the functional form of 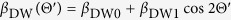 is assumed. The photocurrents of the BT samples under light illumination with a photon energy (*hv*) of 1.97 eV were smaller than the detection limit of our measurement system. The standard deviations of these parameters are shown in parenthesis.
