# Supplementary material for: Giant photovoltaic effect of ferroelectric domain walls in perovskite single crystals
Source: Sci Rep. 2015 Oct 7;5:14741. doi: 10.1038/srep14741 (PMC4595799; doi:10.1038/srep14741)
Supplement: Supplementary Information [file srep14741-s1.pdf]

Giant photovoltaic effect of ferroelectric domain walls in perovskite single crystals (Supplementary Information)

Ryotaro Inoue, Shotaro Ishikawa, Ryota Imura, Yuuki Kitanaka,
Takeshi Oguchi, Yuji Noguchi*[1] and Masaru Miyayama
Dept. of Applied Chemistry, School of Engineering,
The University of Tokyo,
Bunkyo-ku, Tokyo 113-8654, Japan

June 17, 2015

[1]ynoguchi@fmat.t.u-tokyo.ac.jp

Samples

Figure S1a shows the image of a Mn(0.25%)-BT crystal grown by a top-seeded solution growth method. Figures S1b and S1c display the typical PFM images, the in-plane amplitude and the in-plane phase, respectively, of the Mn-BT sample in the $\boldsymbol{J}//[011]$ configuration after the poling treatment. We found a striped shaped 90° domain structure with a domain wall (DW) spacing (W) of $\sim$ 15 μm. We also confirmed that the domains with two different orientations have almost the same volume fraction.

Mn-doping effect on the PV properties

Figure S2a shows the dependences of $|\beta^{\mathrm{bulk}}_{[011]}|$ and $|\beta_{\mathrm{DW0}}|$ on $h\nu$ in the BT samples. We found that the doping of Mn (0.25%) enhances both $|\beta_{\mathrm{DW0}}|$ and $|\beta^{\mathrm{bulk}}_{[011]}|$ by a factor of 50-100 in the $h\nu$ range of 2.5-3.0 eV. In contrast, the absorption coefficients (α) at $h\nu = 3.11$ eV are ~ 300 cm^{-1} (Mn-BT) and ~ 20 cm^{-1} (BT), respectively, showing that the Mn doping increases α by a factor of 15 at most. We can, therefore, say that the Mn doping promotes not only the absorption of under-bandgap lights but also the separation of photogenerated carriers.

In Fig. S2b we plot the corresponding effective electric fields ($|\tilde{E}^{\mathrm{bulk}}_{[011]}|$ and $|\tilde{E}_{\mathrm{DW0}}|$) in the BT samples, where the effective electric field $\tilde{E}$ is defined in the relation of $J_{\mathrm{SC}} = \mathcal{I}_{\mathrm{opt}} \cdot \beta \equiv \sigma_{\mathrm{ph}} \cdot \tilde{E}$. While $|\tilde{E}^{\mathrm{bulk}}_{[011]}|$ remains constant in the $h\nu$ range of 2.5-3.0 eV, the value of $|\tilde{E}_{\mathrm{DW0}}|$ at 2.5 eV is smaller than that at 3.0 eV. This tendency of the effective electric fields is similar to that observed in the Mn-BT samples. We infer that the DW-PV effect in the BT samples due to β_{DW0} is activated at above a threshold of $h\nu$ in the range of 2.5-3.0 eV. The Mn-doping appears to have a lower $h\nu$ threshold of 2.0-2.5 eV.

Photocurrent in the $\boldsymbol{J}//[011]$ configuration

We derive the photocurrent density in the [011] direction in terms of the effective electric field. Figure S2 shows the schematic diagram of the 90° domain structure in the $\boldsymbol{J}//[011]$ configuration. The 90° domain structure can be regarded as the alternate stacking of the two domains in which the spontaneous polarizations, $\boldsymbol{P}_{\mathrm{s1}}$ and $\boldsymbol{P}_{\mathrm{s2}}$, are oriented at angles of +45° and

$-45°$ with respect to the [011] direction. We also define the light-polarization (Θ') as the angle of polarization plane with the [011] direction.

Considering the bulk PV tensor in the BT system given in Eq. (8) in the main text, we can write the [011] components of the photocurrent densities in the single-domain state with $\boldsymbol{P}_{\rm s1}$ and $\boldsymbol{P}_{\rm s2}$ as

$$\begin{aligned} J_{\nearrow} &= \frac{J_2 + J_3}{\sqrt{2}} = \frac{\mathcal{I}_{\rm opt}}{\sqrt{2}} \cdot \left[\beta_{31}\cos^2(2\Theta' + 45°) + \beta_{33}\sin^2(2\Theta' + 45°)\right], \\ J_{\searrow} &= \frac{J_2 + J_3}{\sqrt{2}} = \frac{\mathcal{I}_{\rm opt}}{\sqrt{2}} \cdot \left[\beta_{31}\sin^2(2\Theta' + 45°) + \beta_{33}\cos^2(2\Theta' + 45°)\right]. \end{aligned} \quad (1)$$

Here $J_{\nearrow}$ and $J_{\searrow}$ denote the photocurrent density in the $\boldsymbol{P}_{\rm s1}$ and $\boldsymbol{P}_{\rm s2}$ domains, respectively. We neglect the contribution of β_{15} to the photocurrent, because β_{15} is two orders of magnitude smaller than those of the other components ($|\beta_{31}|$ and $|\beta_{33}|$).

Charge conservation requires a constant photocurrent density even across the 90° DWs. In order to derive the photocurrent density in each domain from Eqs. (S1) and (S2) we first define the [011] component of the effective electric field by using the photoconductivity normalized by light intensity ($\sigma_{\rm ph}/\mathcal{I}_{\rm opt}$) as

$$\begin{aligned} \tilde{E}_{\nearrow} &= \frac{1}{\sqrt{2}} \cdot \left[\tilde{E}_{31}\cos^2(2\Theta' + 45°) + \tilde{E}_{33}\sin^2(2\Theta' + 45°)\right], \\ \tilde{E}_{\searrow} &= \frac{1}{\sqrt{2}} \cdot \left[\tilde{E}_{31}\sin^2(2\Theta' + 45°) + \tilde{E}_{33}\cos^2(2\Theta' + 45°)\right]. \end{aligned} \quad (2)$$

Here, $\tilde{E}_{31}$ and $\tilde{E}_{33}$ are the effective electric fields corresponding to β_{31} and β_{33}, respectively, where $\beta_{i\lambda} \equiv (\sigma_{\rm ph}/\mathcal{I}_{\rm opt})\tilde{E}_{i\lambda} \quad ; i\lambda = 31, 33$. The overall photovoltage $\tilde{V}_{\rm ph}$ is obtained by integrating the effective field in each domain followed by adding the net photovoltage $\tilde{V}_{\rm DW}(\Theta')$ at the 90° DW:

$$\tilde{V}_{\rm ph} = N \cdot \left[W(\tilde{E}_{\nearrow} + \tilde{E}_{\searrow}) + 2\tilde{V}_{\rm DW}(\Theta')\right] \quad (3)$$

where L denotes the sample length and $N \equiv L/2W$ the number of the alternate stacks of the 90° domains between electrodes. We can rewrite the photocurrent density in the [011] direction ($J^{\rm bulk}_{[001]}$) using $\tilde{V}_{\rm ph}$ as

$$J^{\rm bulk}_{[011]} = \sigma_{\rm ph}\frac{\tilde{V}_{\rm ph}}{L} = \sigma_{\rm ph}\left(\frac{\tilde{E}_{\nearrow} + \tilde{E}_{\searrow}}{2} + \frac{\tilde{V}_{\rm DW}(\Theta')}{W}\right). \quad (4)$$

The first term in the right-hand side stems from the bulk PV effect that is identical to Eq. (2) in the main text. The second term is identical to $\tilde{E}^{\text{bulk}}_{[011]}$, which consists of the screened electrostatic potential step ($\Delta\Phi^{\text{sc}}_{\text{DW}}$) and the local PV component in the DWs.

Effective electric field representing the bulk PV effect

As expressed in Eq. (4) in Method the bulk PV tensor is defined using the photocurrent density and the light intensity. The bulk PV effect can also be described in terms of an effective electric field. Here, we consider a non-centrosymmetric crystal under uniform, continuous light irradiation, where photoinduced carriers are generated homogeneously throughout the bulk crystal and a diffusion current is not present. A photocurrent arising from the bulk PV effect corresponds, therefore, to a drift current, which originates from the effective electric field.

A significant asymmetry in the photogenerated carrier dynamics due to spatial symmetry breaking induces the effective electric field. The densities of the photogenerated carriers, i.e., electron density (n) and hole density (p), are determined by the equilibrium between the rates of generation and recombination of the carriers. Using the mobilities of the photogenerated electron (μ_{e}) and hole (μ_{h}), we can express photoconductivity (σ_{ph}) as

$$\sigma_{\text{ph}} = |e|(p\mu_{\text{h}} + n\mu_{\text{e}}), \tag{5}$$

where $|e|$ is the elementary charge. As shown in Table II, the components of the bulk PV tensor (β_{31} and β_{33}) reflect the anisotropy of the bulk PV effect while σ_{ph} was found to be isotropic. The photogenerated carrier densities have, therefore, an anisotropy with respect to $\boldsymbol{P}_{\text{s}}$ in the bulk PV effect.

Focusing on the photocurrent in the $\boldsymbol{P}_{\text{s}}$ direction we divide the photogenerated carriers into two groups according to each respective drift velocity in the direction *same* or *opposite* to the $\boldsymbol{P}_{\text{s}}$ vector: the electron densities of n_+ and n_- and the hole densities of p_+ and p_-, where the subscript represents the same (+) or the opposite (−) direction. The net density of the photogenerated electron or hole, which constitutes the photocurrent in the $\boldsymbol{P}_{\text{s}}$ direction, is given by $(n_+ - n_-)$ or $(p_+ - p_-)$. The short-circuit current densities $J_{\text{SC}(+)}$ and $J_{\text{SC}(-)}$ are ascribed to (n_+, p_+) and (n_-, p_-), respectively, which are expressed as

$$J_{\text{SC}(\pm)} = |e|(p_\pm \bar{v}_{\text{h}} + n_\pm \bar{v}_{\text{e}}). \tag{6}$$

We define $\bar{v}_{\rm h}$ and $\bar{v}_{\rm e}$ as the averaged drift velocities projected onto the $\boldsymbol{P}_{\rm s}$ direction, where the average is taken over a solid angle of 2π. The net photocurrent density ($J_{\rm SC}$) is given by the difference between $J_{\rm SC(+)}$ and $J_{\rm SC(-)}$:

$$J_{\rm SC} \equiv J_{\rm SC(+)} - J_{\rm SC(-)} = |e| \left[(p_+ - p_-)\bar{v}_{\rm h} - (n_+ - n_-)\bar{v}_{\rm e} \right]. \tag{7}$$

In the centrosymmetric crystals, $J_{\rm SC}$ becomes zero because $p_+ = p_-$ and $n_+ = n_-$.

The asymmetry in the carrier dynamics due to spatial symmetry breaking is closely related to the photoexcitation process. Here we adopt a simplified model in which a single parameter (γ) correlates $n_\pm$ and $p_\pm$ with n and p as $n_\pm = n(1 \mp \gamma)/2$ and $p_\pm = p(1 \pm \gamma)/2$. We obtain the following relation using $\tilde{E}^{\rm bulk}(= J_{\rm SC}/\sigma_{\rm ph})$:

$$(p\mu_{\rm h} + n\mu_{\rm e})\tilde{E}^{\rm bulk} = \gamma(p\bar{v}_{\rm h} + n\bar{v}_{\rm e}). \tag{8}$$

Estimation of screening of $\Delta\Phi_{\rm DW}$

We estimate the screening of $\Delta\Phi_{\rm DW}$ based on the carrier-density dependence of chemical potential. Considering that the majority carrier is hole in the Mn-BT crystals annealed under an oxidized atmosphere, we can neglect the screening effect by electrons. The electrostatic potential step ($\Delta\Phi_{\rm DW}$) causes the photogenerated holes to accumulate on one side of each DW and to escape from the other. The resultant distribution of the hole density provides a partial screening of $\Delta\Phi_{\rm DW}$.

According to Boltzmann statistics, the difference in chemical potential of the hole system ($\delta\zeta_{\rm h}$) is expressed as $\delta\zeta_{\rm h} = k_B T \ln(p/p_0)$, where p_0 and p denote the hole densities under dark and light conditions, respectively, and $k_B T$ the thermal energy at room temperature ~ 26 meV. The experimental data on the relative change in $\sigma_{\rm ph}$ give an approximate estimate of $p/p_0 \sim 10^3$ under $\mathcal{I}_{\rm opt} = 1$ W/cm^2, leading to a $\Delta\Phi_{\rm DW}$ of ~ 180 meV. We can regard this $\Delta\Phi_{\rm DW}$ value as the upper limit of the screening of $\Delta\Phi_{\rm DW}$, because the change in p caused by the redistribution around the 90° DWs is smaller than p/p_0. Thus, the electrostatic potential step involving the screening effect, i.e., the screened electrostatic potential step $\Delta\Phi_{\rm DW}^{\rm sc}$ is estimated to be $\Delta\Phi_{\rm DW}^{\rm sc} = \Delta\Phi_{\rm DW} - \delta\zeta_{\rm h}/|e| \sim 50$ mV at least, which is still larger than the experimental value of $\tilde{E}_{\rm DW0}W \sim 37.5$ mV.

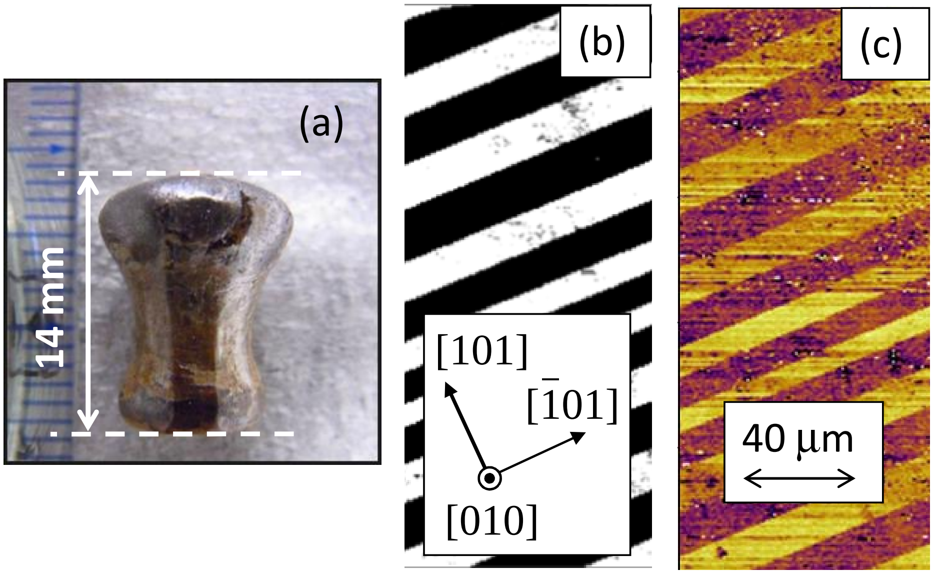

Figure S 1: (a) A Mn(0.25%)-BT crystal grown by a top-seeded solution growth method. Typical PFM images of the 90° domain structure observed in the $\boldsymbol{J}//[011]$ configuration in the Mn-BT samples: (b) in-plane amplitude and (c) in-plane phase.

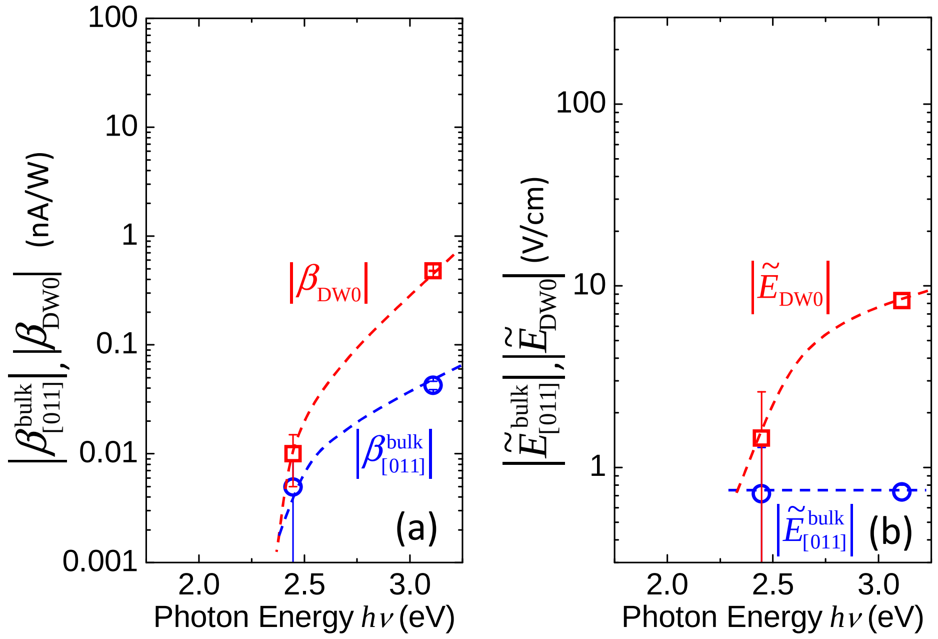

Figure S 2: Comparison of the bulk PV effect with the DW-PV effect in the BT samples: (a) $|\beta^{\rm bulk}_{[011]}| \equiv |\beta_{31} + \beta_{33}|/2\sqrt{2}$ and $|\beta_{\rm DW0}|$ and (b) the corresponding effective electric fields ($|\tilde{E}^{\rm bulk}_{[011]}|$ and $|\tilde{E}_{\rm DW0}|$). The broken curves are guides to the eye. The standard deviation of each data point is shown as an error bar.

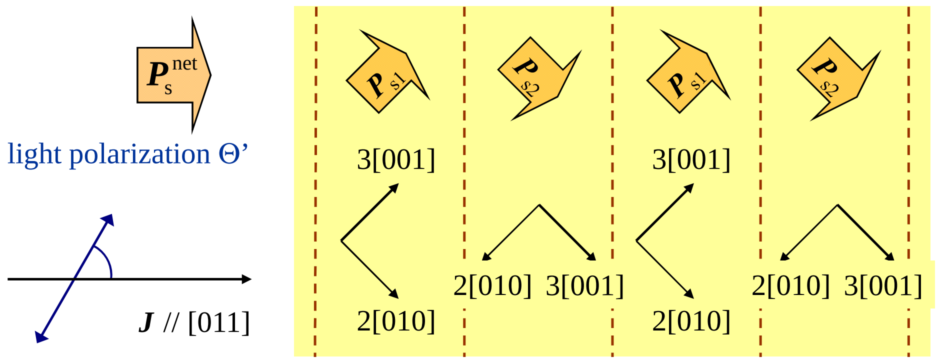

Figure S 3: Schematic diagram of the 90° domain structure in the tetragonal BT system. The crystal axes together with the orientation of spontaneous polarizations are shown in each domain.
